# Lithium Monitoring in Adult Community Mental Health Patients: Evaluating Current Practices in Al Ain, United Arab Emirates

**DOI:** 10.1192/bjo.2025.10491

**Published:** 2025-06-20

**Authors:** Mouza AlDhaheri, Salma AlAzeezi, Fatima AlShanqiti, Taibah AlMadhaani, Syed Fahad Javaid

**Affiliations:** 1 Behavioral Sciences Institute, Al Ain Hospital, Al Ain, UAE; 2 Department of Psychiatry, College of Medicine and Health Sciences, United Arab Emirates University, Al Ain, UAE

## Abstract

**Aims:** Lithium is a well-established mood stabiliser used for conditions such as bipolar disorder, mania, and depression. Given its narrow therapeutic index, regular monitoring is essential to prevent toxicity, which can cause confusion, seizures, coma, or death. This audit evaluated long-term lithium monitoring compliance for adult community-based patients at the Behavioural Science Institute, Al Ain Hospital, United Arab Emirates. We assessed adherence to local hospital guidelines, which align with The National Institute for Health and Clinical Excellence (NICE) Guideline CG185, recommending six-monthly checks of serum lithium levels, thyroid function, renal function, calcium levels, and BMI, with some patient groups requiring more frequent testing notably older adults, those taking medications interacting with lithium, and patients at risk of kidney, thyroid, or electrolyte abnormalities.

**Methods:** This retrospective audit reviewed electronic case notes of 38 patients on long-term lithium therapy under community mental health services from January 2018 to January 2019. A questionnaire was developed to capture the necessary information anonymously. Data collection occurred between March and May 2024.

**Results:** Of the 38 patients, 20 (53%) were male, with an age range of 20–64 years and a mean age of 35.2 years. The most common diagnosis was bipolar disorder, which accounted for 44.7% of cases, followed by schizoaffective disorder at 18.4% and major depressive disorder at 10.5%. Regarding six-monthly monitoring, serum lithium levels were measured in 25 patients (65.8%), with documented reasons provided for only six of the 13 patients who did not undergo testing. BMI was monitored in 36 patients (94.7%), while thyroid function tests were conducted for 31 patients (81.5%). Renal function was assessed in 27 patients (71%), with urea and electrolytes checked in 32 patients (84.2%). Notably, serum calcium levels were measured in only three patients (7.8%), highlighting a significant gap in monitoring adherence.

**Conclusion:** The audit revealed considerable inconsistencies in lithium monitoring, particularly in the assessment of serum lithium and calcium levels. To address these gaps, we recommend enhancing staff training on lithium prescribing and monitoring protocols to ensure adherence to guidelines. Optimising electronic prescribing systems to generate automated reminders may improve compliance, while better documentation practices are needed to ensure that any deviations from standard monitoring are appropriately justified. A re-audit will be conducted one year after implementing these measures to evaluate their effectiveness.

No financial sponsorship has been received for this evaluative exercise.

